# Men’s perspectives on male participation in antenatal care with their pregnant wives: a case of a military hospital in Lusaka, Zambia

**DOI:** 10.1186/s12913-019-4294-8

**Published:** 2019-07-08

**Authors:** Hamalambo Muloongo, Doreen Sitali, Joseph Mumba Zulu, Alice Ngoma Hazemba, Oliver Mweemba

**Affiliations:** School of Public Health, University of Zambia, PO Box 50110, Lusaka, Zambia

**Keywords:** Men’s perspectives, Male participation, Antenatal care, Military hospital

## Abstract

**Background:**

Male partner participation in antenatal care (ANC) is important and contributes to better maternal and neonatal birth outcomes. Studies have been conducted to explore male participation in ANC and barriers to participation. However, these studies have been conducted in the general population and not the military settings, which are gendered institutions. This study aimed to explore the perspectives of male participation in ANC in a military setting.

**Methods:**

A qualitative case study approach using convenient sampling was used to enlist sixteen (*n* = 16) military men whose partners or wives were attending ANC. In-depth interviews were conducted with participants to get their perspectives on their participation in ANC. The interviews were transcribed verbatim and codes, categories and themes were generated from the data. Data analysis was done manually and was guided by thematic framework analysis approach. We designed a table which listed all emerging themes, categories and sub-themes.

**Results:**

Participants were aged 27–45 years and some attained tertiary education. Five themes emerged to explain the perspectives of male participation in ANC. Men’s roles were perceived to be limited to provision of appropriate food and supplies, physical and emotional support. Generally, ANC attendance was considered a woman’s private activity because even health care providers were mostly female. However, the desire to have a healthy baby prompted many to seek information on ANC. On the other hand, priority given to couples attending ANC and the need to be part of the decision making motivated some to participate. For the participants in this study, military operations, fear of being tested for HIV and the belief that presence of men in ANC interferes with care made them shun the services.

**Conclusion:**

Lack of awareness on the importance of male participation in ANC impacted on the understanding of access and use of services by participants. The study has practical implications in the military institution to formulate policy on male participation in ANC to improve maternal and newborn health outcomes as well as support staff who have to attend to their pregnant wives or partners.

## Background

Male partner participation in antenatal care services is vital if pregnant women are to fully utilize and benefit from the services offered [1]. Husbands, particularly in African countries, play a pivotal role in decision-making within a home, and are often the breadwinners. Therefore, establishing their participation and support for sexual reproductive health service utilization is critical [2–5].

Researchers have advanced various definitions of male partner participation in sexual reproductive health. Some define it as the process of social and behavioural change that is needed for men to play more responsible roles in maternal health care with the purpose of ensuring women’s and children’s wellbeing [6] or men accompanying their spouses, providing social-economic support and using family planning as well as HIV prevention measures [7]. Others have defined it as men attending antenatal health care visits, birth plans, encouraging exclusive breast feeding and immunization for their children [8]. In this study, male partner participation entails a male partner or husband accompanying his wife or female partner to Antenatal Care (ANC), providing social economic support and ensuring that all recommendations made at ANC are observed to safeguard the wellbeing of the couple and the baby.

It is noted that, sexual and reproductive health programmes and services have focused primarily on women. Consequently, men have often lacked information to make informed decisions about healthy behaviour and the roles they might play in promoting overall family health [9]. Men are encouraged to participate in sexual reproductive health to enhance utilization of services and improve the health of the entire family [10]. When male partners do not participate in antenatal care, chances of women and men fully adhering to antenatal clinic interventions diminish tremendously [11–14].

Male partner participation in antenatal care services is low in most Zambian military cantonments. For example, at the site where this study was conducted, only one in twelve pregnant women attended antenatal care with the man responsible for the pregnancy during the period 2011 to 2013 [Hospital Health Management Information System, (2013), Unpublished]. Several studies have been conducted in Zambia and other countries to find out levels of male participation in antenatal care and barriers to male participation. However, the studies in Zambia have been conducted in the general population leaving out military settings.

The study objectives were: describing the roles of men in antenatal care; understanding their motivation for attending antenatal care and reasons for failure to do so, and to describe masculinity in the context of male participation in antenatal care activities. These objectives take into account the fact that military institutions, in terms of their organisation, their personnel, their activities and their effects, are gendered institutions. As gendered institutions with their own specific structures and organizational cultures, militaries shape their members’ behaviors through the construction and reproduction of norms and the development of rules and policies governing individual activities. As gendered institutions, militaries shape the daily lives and lived experiences of those working within or alongside them [15].

No similar study has been conducted and published in Zambia on male participation in antenatal care in a military institution due to its restricted nature. Therefore, evidence from this research will guide the practice of population-based service provision in such settings.

## Methods and materials

### Study design, site and population

This was a qualitative study using case study approach. The case was a military cantonment in Lusaka Zambia. The research approach was helpful in getting a detailed insight of men’s perspectives concerning participation in ANC.

The study site was a hospital facility in a military cantonment in Lusaka, Zambia. The catchment population, including the civilian population surrounding the cantonment, was 20,000 (Hospital Health Management Information System; 2013, Unpublished). Most of the residents were in formal employment. Other economic activities included trading and other small and medium enterprises. The Zambia Army in cooperation with the Ministry of Health, and some Non-Governmental Organisations (NGOs) provide the health services for this population. The hospital provides preventive, diagnostic and curative health care services, as well as maternal, newborn and child health services. The hospital has a bed capacity of thirty-three. The study site was purposively selected because there were pregnant women attending antenatal care services and male partner involvement in antenatal care was low, one in every twelve (Hospital Health Management Information System; 2013, Unpublished).

The study participants comprised military men who resided or worked in the military cantonment and whose wives or partners were pregnant and attending antenatal care at the military hospital at the time of data collection. Sixteen military men (*n* = 16) participated in the study and the rank structure included Major, Captain, Lieutenant, Warrant Officer, Staff Sergeant, Sergeant and Corporal. The overall aim for the study was to explore military men’s perspectives on their participation in antenatal care with their pregnant wives or partners. The study objectives were: to describe the role of men in antenatal care; to understand the motivating factors for attending antenatal care, and to explore masculinity in the context of male participation in antenatal care activities.

### Sampling procedure

The study conveniently sampled military personnel whose wives attended antenatal services at the hospital facility in the military cantonment. At the health facility, we provided an opportunity to whoever came for antenatal care to participate in the study until data saturation was reached. Each day, the first two people to attend antenatal care were requested if they could participate in the study until the sample was reached. Only two were targeted each day to allow the researchers review the information from the interviews. Participants (military men) who attended antenatal clinic with their wives were enlisted directly at the Camp Hospital during antenatal sessions. Pregnant women who were not accompanied by their male partners were offered an information sheet and consent form to invite their male partners to participate in the study. Sixteen participants were recruited in the study. Three were recruited directly while thirteen were recruited through their expectant wives. Of the over thirty information sheets handed out, only thirteen participants turned up while others were reported to be away on military duties.

### Data collection, processing and analysis

In-depth interviews (IDIs) were used to collect data. The interviews were done face to face with men who participated or not in antenatal care with their wives or partners. An interview guide with open-ended questions was used to administer the interviews. It was designed to probe on roles husbands play during antenatal care, what motivates men to participate or not in antenatal care and description of masculinity in the context of antenatal care. Interviews were conducted from the hospital premises, from the participants’ offices and from participants’ homes. On average, each interview lasted thirty minutes. All interviews were audio recorded and all participants gave a written informed consent to the use of a recorder. Furthermore, observations were made and noted during the interviews and throughout data collection. Data saturation was reached when no more information was elicited new themes emerged during data analysis.

Data preparation and organization was done immediately after each interview. Audio files and notes were adequately labeled for easy management. Labeling involved using codes to prevent the revelation of participants’ identity. Interviews or sections thereof done in local languages were translated into the English language.

We used the framework approach to guide data analysis. The approach is gaining popularity among health care researchers and was developed by Jane Ritchie and Liz Spencer in the late 1980s [16, 17]. Audio files were transcribed verbatim. The transcripts were read several times to gain an understanding of the data. We identified the thematic framework through initial coding of data which was followed by indexing, involving the application of analytical framework to data. Indexing is the systematic application of codes from the agreed analytical framework to the whole dataset [17]. We applied the working analytical framework to subsequent transcripts using the existing categories and codes. This enabled us find patterns in the data.

Data analysis was done manually. This was done by designing a table that listed all identified codes which participant brought out during interviews. Finally, we searched for patterns, associations, concepts, and explanations in the data. We used a framework matrix table in which individual participants (cases) were included in the rows, codes which we identified from the data were included in the columns and when summarized were placed in the matrix cells. This provided a structure into which we could systematically reduce the data, in order to analyze it by case and by code [17]. Using the framework matrix, we looked for themes and patterns across datasets that are important to the description of the concept of male partner participation in antenatal care with their expectant partners. HM and OM independently did the coding of the transcripts which were later compared and consensus reached. Later the other researchers ANH, JMZ and DS scrutinized and verified the coding.

Table 1 shows the sub-themes, categories, and themes generated after analysis of data.

**Table 1:** Selected sub-themes, categories and themes

Sub-themes	Categories	Themes
Provision of necessities during pregnancy	Views on roles of men in antenatal care	Roles of men in antenatal care
Acquiring information on pregnancy and needed care		
Providing physical and emotional support to wife		
Knowing what pregnant women go through	Views on meaning of men attending antennal clinic	Meaning of men attending antenatal clinic
Expression of love and care		
Collective responsibility		
Opportunity for learning		
Deprivation of privacy		
Barrier to appropriate care		
Knowledge of importance of attending ANC	Enabling factors	Motivation for men to attend or not attend antenatal care with their wives
Desire to get information about pregnancy and care		
Desire to have a healthy mother and baby		
Privileges given to those who attend as couples		
Desire to be part of decision making		
Desire to be a responsible father		
Military operations	Deterring factors	
Not knowing that men also need to attend ANC		
Belief that presence of husband interferes with care		
Fear of HIV test		
Feminine environment	Views on masculinity	Masculinity
Female health workers		

### Ethical considerations

We sought for ethical clearance from ERES Converge ERB (Ref. No. 2014-May-024). We also sought written and informed consent from all the study participants. Participants were given identifiers to ensure privacy and confidentiality. Only the research team had access to the information collected and will be destroyed after 5 years.

## Results

### Participants’ information

All the participants were male military personnel whose wives were pregnant and attending antenatal care at the time of the study. They were aged from twenty-seven to forty-five years. The level of education ranged from grade nine to tertiary level. The length of marriage ranged from eight months to nineteen years. The number of children ranged from zero to six.

Table 2 shows the social demographic characteristics of the study participants.

**Table 2:** Social-demographic characteristics of study participants

Age		Educational level		Duration of marriage		Number of children	
Category	Number	Category	Number	Category	Number	Category	Number
25–30 Years	02	Grade 9	04	0–5 Years	04	0–1	03
31–35 Years	02	Grade 10	02	6–10 Years	04	2–3	08
36–40 Years	03	Grade 12	05	11–15 Years	05	4–5	04
41–45 Years	09	Diploma	05	16–20 Years	03	6–7	01
Total	16	Total	16	Total	16	Total	16

Five themes emerged; role of men in antenatal care, perception on participation in antenatal care, motivation to attend antenatal care, deterrents to participation in antenatal care and experiences during participation.

### Role of men in antenatal care

We asked the participants to explain the role husbands play in antenatal care. Participants highlighted that men’s role was limited to provision of necessities, such as appropriate food and other requirements during pregnancy, acquiring information on pregnancy and the needed care as well as provision of physical and emotional support to their wives during pregnancy. The following subthemes describe the different roles with participant quotes:

#### Provision of requirements

Some participants narrated that it was the role of men to provide nutritious foods, appropriate clothing, and shelter for their pregnant women for them to remain healthy and comfortable during pregnancy. No participant spoke to the contrary, but a few were silent on this matter. One participant said:

*There are certain requirements that we need to provide during pregnancy as well as during labour…you know…when a woman is pregnant there are certain things she needs…she needs adequate food and she may easily get tired so you need to help her out in home chores*. (Participant No. 10, usually attended ANC).

#### Acquiring information on pregnancy and care

Most of the participants highlighted that it was the husband’s role to acquire information on pregnancy and care to promote a safe pregnancy. It was emphasized that the husbands needed to learn how to care for their pregnant wives and to know the danger signs during pregnancy and what to do in case of an emergency. The information could also be used to monitor the health of both the mother and the baby. A man who regularly attended ANC explained:

*Men should be keen so that they learn more about antenatal care so that they will in turn be able to take care of their wives even when the medical people are not there.* (Participant No. 15, a married man who regularly attended ANC).

### Perception of men participating in ANC with their wives

Some participants perceived male attendance at antenatal clinic to be an expression of love and care. On the other hand, some participants felt that a husband attending antenatal care with the wife would deprive the wife of the privacy needed during the physical examination. The following subthemes describe this perspective:

#### Expression of love and care

It was generally expressed by most participants that men’s attendance at antenatal clinic was an expression of love and commitment as well as physical and emotional support for the pregnant wife. It was emphasized that when a pregnant mother experiences love from her husband, both her wellbeing and that of the unborn child are enhanced tremendously and that the opposite resulted in adverse outcomes such as miscarriages and abortions. A newly married man who regularly attended ANC explained:

*Ah…to me, going with my woman to antenatal care is a sign of commitment…just to know the general wellbeing…some of the complications women face during pregnancy are as a result of negative feelings because maybe the wife feels neglected by the husband…you know even the miscarriage issue…if the man is not contributing positively while the woman is pregnant, such happens because it’s a trauma* (Participant No. 2, a newly married man who regularly attended ANC).

On the other hand, some participants who never participated in antenatal care were of the view that physical and emotional support should be provided at home and not at antenatal clinic.

*You know…like at home…I can just say assisting her because she becomes tired of which she can’t do some house chores…you have to be there helping* (Participant No. 9 never attended ANC)*.*

#### Deprivation of privacy

For some participants, attending ANC as men was considered as infringing on the privacy of pregnant women. A married participant who never attended ANC had this to say:

*…is it not confidential? Confidential in terms of the people who are attending to her or is it not restricted? The way women explain to us, as if me I am not supposed to be there. So I don’t think it is necessary for me to go there…but I can be there quite alright…waiting for her to be screened and whatever is happening…but not me being where she is screened from.* (Participant No. 9, never attended ANC).

### Motivation to attend antenatal care

Regarding the motivation to attend ANC, participants highlighted elements such as the desire to get information about pregnancy and care; the desire to have a healthy mother and baby; the privileges couples receive; and the desire to be part of decision-making. The following subthemes describe these motivating factors with participant quotes:

#### Desire to have a healthy mother and baby

Regarding the desire to have a healthy mother and the baby, the participants who attended ANC highlighted that the information they received helped to improve and maintain the health of the unborn baby and the mother.

*…what motivates me most is that I want to see that the baby is born in good health…even afterwards not to stop because the issue of care should not end at delivery. I want a situation where even I as a father I get the baby to under-five clinic and my wife remains doing other chores.* (Participant No. 7, expecting first child and missed the first ANC visit due to military commitment).

#### Privileges given to couples

Those who attend as couples received special treatment such as being attended to first.

*Ah, what motivates me to come with my wife to antenatal care is the privilege that they are giving. Like in some clinics they always request for those who come as a couple to be attended to first…that thing has motivated me …my wife does not delay when she comes for antenatal care*. (Participant No. 8, regularly attended ANC).

#### Desire to be part of decision-making

Making a timely decision during pregnancy and at child birth was considered as important. A participant whose wife lost the previous baby explained:

*When she gave birth, the baby died…and we discovered that the child was too big…weighed 4kg and she gave birth through the normal way and they were saying that she should have delivered by caesarian section. From that day, I got interested. I said if these people cannot make the decision I have to make it myself and I have to be involved*. (Participant No. 15 regularly attended ANC)

However, some men who never participated in antenatal care were of the view that they did not necessarily have to attend ANC to be part of the decision-making process. They argued that where their input was required, they could be invited to the clinic and would oblige.

…*eh…me what I know from way back is that antenatal is for women…you see…but then when there is a problem they call you as a man so that you are part of the solution* (Participant No. 9 aged 37, years never attended ANC).

### Deterrents to attending antenatal care

We asked the participants to explain what prevented them from attending antenatal care with their spouses. The responses included: military operations; lack of awareness that men needed to attend; belief that women and or health workers would be uncomfortable; and fear of an HIV test.

#### Military operations

For some participants, it was inevitable for them not to attend ANC because they had to respond to instructions and attend military operations even when their wives were pregnant. They had various operations such as local safeguard and international duties under the auspices of the United Nations Organization. One participant explained:

*Being a military man, most of the time I am out on operations hence I could not come with my wife those times that I was away.* (Participant No. 6, attended ANC).

#### Lack of awareness of men’s involvement in ANC

Regarding men attending ANC, it was generally observed that men knew that it was an activity for women alone.

*Okay this time when she fell pregnant and came to the antenatal clinic that is when she explained to me that we needed to come together*. (Participant No.4, never attended ANC).

#### Fear for an HIV test

Some participants feared testing for HIV with their spouses thinking it could be a source of misunderstanding for the couple. They feared because most of them would get into sexual relationships while they were engaged in military operations. Therefore, they feared to test with their spouses in case of an HIV positive result. A participant who only attended ANC once and was scared but received encouragement from his superior explained:

*At first I was scared…I thought that maybe they would start asking me about my status [HIV status] so I was like…let me just wait…at first she told me to come to the hospital but I was scared until I was encouraged by my boss at work*. (Participant No. 5, attended ANC once).

*I was against that [going with my wife for ANC] but now I can encourage my friends to go there so that one, they can know their status [HIV], two, they should know what happens before birth* (Participant No. 4 never attended ANC).

#### The belief that presence of the husband would interfere with care

Some men thought that pregnant women would be uncomfortable if health workers attended to them in the presence of their husbands and that health workers would not be free to provide the services in the presence of men.

*You see…there are some issues….whereby when the doctor is talking to the woman, she may not be comfortable when the husband is present and maybe there is something that the doctor may also not be confortable asking the woman certain questions in the presence of her husband. So it is better to stay outside*. (Participant No. 1, regularly escorted wife to ANC but always remained outside).

### Experiences during participation in ANC

For this study, the experience of men attending ANC was described in the context of the activity being dominated by females especially that the health workers were also female. These perspectives are described in the following subthemes.

#### Feminine environment

While some participants felt uncomfortable to be in the presence of many pregnant women at ANC, they still attended because it was important for them to do so. A participant who attended ANC by invitation by the nurse described his experience in the following quote:

*Ah…I was feeling shy until my wife was called inside by the nurse…the nurse also said you call your husband but I was feeling shy to go inside…I thought maybe there was…those women could look at me that there is a problem maybe that is why he has come.* (Participant No. 5, attended ANC once on invitation by a nurse).

#### Presence of female health workers

Most of the men said it was normal for female health workers to attend to them because they were trained in that field. They also explained that women were the ones who fell pregnant and therefore taught better about issues of pregnancy, unlike men who could only give hearsay information. One participant even emphasized that some instructors in military courses were female and it was normal.

*It is just okay…it is the same when you go for most of these military courses…you find women instructors teaching…so it is just okay as long as you get the knowledge* ( participant No. 12, never attended ANC).

However, another participant was generally uncomfortable with the presence of female health workers attending to him, although his concern did not relate to antenatal care.

*Ah…me…of course you may feel uncomfortable but like I said earlier on, when it comes to issues of health, they are actually more important than even having money. However, if there can be a situation where they can be put two [a male and a female] it can be better. In fact, it is not only at antenatal clinic, but also at all departments. For example, when I went for circumcision, the first person I saw was a man. He then directed me to a room where I found women and that somehow disturbed me…being worked on by women.* (Participant No. 7)

## Discussion

The aim of this study was to explore men’s perspectives and experiences of male involvement in ANC. The major thematic areas were: the understanding of the role of men in ANC, perspectives of men attending ANC, motivating factors to male attendance in ANC, factors that deter men from attending ANC, and experience s of men who attended ANC.

For this study, the results showed that ANC attendance is considered as a women’s activity especially that in Zambia the services are predominantly provided by female health workers. This perception is not unique to the studied population in Zambia because it is associated with femininity in similar settings and it has attracted a global debate in the context of safe motherhood [6, 18, 19].

Therefore, regarding the role of men in ANC, studies have shown that male involvement in safe motherhood has become a topical issue since the concept of reproductive health and rights was adopted at the International Conference on Population and Development (ICPD) in 1994. During the ICPD conference it was discovered that the implications of involving men in maternal health services such as ANC is deeply rooted in the way in which society defines gender roles and responsibilities [20–22]. However, the importance of involving men in safe motherhood activities such as ensuring well-being and survival of mothers during pregnancy and childbirth can-not be over emphasized. The participants in this study were aware that their involvement in ANC accorded them an opportunity to learn about pregnancy and the care needed to ensure good health of the mother and the unborn baby and for improved maternal and newborn health outcomes. As noted in the results of this study, losing a baby as a couple is devastating and therefore, the need for men to be part of the decision making in childbirth was identified as critical. Studies in similar settings have shown that involving men in ANC leads to improved birth preparedness and complication readiness [6, 23]. Some of the elements of birth preparedness include among others knowledge of danger signs during pregnancy and child birth, plan for where to give birth, who to attend to child birth, transportation and sourcing for money. In this setting, men are influential in providing not only money but other necessities that might be required at child birth and hence involving them at ANC provides an opportunity for them to plan early for child birth and avoid delays.

Despite efforts in involving men during pregnancy and child birth, deterring factors remain. In this study some of the factors identified that hindered men to attend ANC were unique to the population studied. Call for duty for military operations either locally or internationally may be beyond the control of men in uniform. However, the results of this study may inform policy when assigning duties to men in uniform. No study was identified in similar settings to draw lessons on how best the management can deal with this situation. In this instance, they may understand the value of allowing men to be part of child birth for their spouses for a defined period. As observed, it was evident that in the Zambia Army, issues of the health of troops and their families were considered to be very important. Similarly, in other military settings such as the United States of America, a study revealed that if a member of the military wing was distracted about his or her family’s quality of life, then his efficiency and productivity were greatly compromised [24].

However, the participants were not immune to other factors that hinder men from participating in ANC such as fear of being tested for HIV. In Zambia, couple counseling and testing is encouraged as an entry point for treatment and elimination of mother-to-child transmission of HIV [25] and is a strategy other countries in sub-Saharan Africa are promoting among men [26–28]. However, they are not forced to take a test and know their results. As indicated by participants, men who spend a long time away from their spouses are prone to extra marital affairs and potentially risk practicing unprotected sex because of the extended period of absence from their spouses. In this regard, attending ANC might accord them an opportunity to talk about their fears, challenges and to get information on how they can protect themselves from contracting HIV.

The knowledge about the importance of male involvement in safe motherhood is not widespread in this and similar settings [28, 29]. Awareness programs to promote male involvement in activities such as ANC, child birth has been implemented in some settings. Some participants in this study were genuinely not aware that they could attend ANC and that is why they thought that it was a women-oriented environment. A military setting in Zambia is male dominated and this could explain the status quo of considering that any program for pregnant women could not be associated with men. Considering that some participants said it was normal for them to be attended by female health workers because they were qualified can be strengthened so that the community could build the confidence in this area. There is need to dispel the notion that fellow women were better placed to teach about pregnancy since they experience child birth as well. In military settings, the issue of gender in ranks is not very pronounced where superiority is the key regardless of whether the person is female or male, and younger or older. Riding on this could be an advantage to use women and men to conduct awareness programmes as well offer services in all the facets of health care.

## Conclusion and implication for safe motherhood

The aim of this study was to explore the perspectives of men regarding their participation in ANC. The findings showed that military men were generally willing to participate in ANC but lacked knowledge on their relevance which had a bearing on how they perceived the roles and how to deal with a female dominated environment. It is important to note that the study has practical implications at Command level, whose responsibility is to formulate policy on male participation in ANC in order to improve maternal and newborn health outcomes. Particularly, the Military Medical Services Branch should plan and implement activities that are responsive to the expectations of both men and women.

## Recommendation

The methodological approach describes a systematic process of conducting the study in a military setting. Therefore, the study sets the stage for quantitative researchers to conduct studies with larger samples in order to generalize the findings to specific populations. Furthermore, identified gaps, such as comparing first child pregnancy and later pregnancies and sampling stratified by age and employment status should be considered.

## Limitations and strengths of the study

The sampling method, [purposive sampling] is generally criticized by quantitative researchers. The method of recruiting participants, where the clinic was used as the start-point, could have missed some women and men who did not attend antenatal clinic at that time. There could have been some desirability bias in the way participants responded to questions seeing that one of the researchers was a superior officer in the military. Furthermore, the study failed to compare the level of participation between first child pregnancy and later pregnancies.

However, the study revealed the perspectives of men in uniform concerning their participation in ANC. This will help health workers to address the relevance of male participation in ANC regardless of the settings such as the military.

## Data Availability

The datasets generated and analysed during the current study are not publicly available due to risk of compromising individual privacy but are available from the corresponding author on reasonable request.

## Abbreviations

AIDS: Acquired Immune-Deficiency Syndrome
ANC: Antenatal Clinic or Antenatal Care
HIV: Human Immuno-deficiency Virus
HMIS: Health Management Information System
ICPD: International Conference on Population and Development
IDIs: In-Depth Interviews
IRB: Institutional Review Board
PMTCT: Prevention of Mother to Child Transmission (of HIV)
USA: United States of America
WHO: World Health Organisation
